# On the Introduction of the Ligature

**Published:** 1805-07-01

**Authors:** 


### *To the Editors of the Medical and Physical Journal.*

GENTLEMEN,

I MET, by accident, a few days since, with the 75th number of your Journal, and was, I confess, rather surprised at the contents of a paper, signed Chirurgicus, in which the author accuses Mr. Symmons of ignorance, in ascribing to Parè the first introduction of the ligature in amputations.

The first point, perhaps, that would strike a common reader is, the mention he makes of the incursions of Henry the Fourth into Italy; whereas that great monarch never, either before or after his accession to the crown of France, penetrated beyond some towns of Savoy, or ever sent an army into Italy; and, on further inquiry, we shall find, that even this expedition into Savoy did not take place till the year 1600*, ten years after the death of Parè, who died, at a very advanced age, in 1590†, the year after Henry came to the crown, after having been first surgeon to the four preceding kings. Not that I would mean, by this, to infer, that Parè had not, many years before this period, served in Italy as surgeon in the armies of Francis the First and his successor, and probably seen the ligature used to stop hæmorrhage in gun-shot or other wounds. That some of his countrymen also, through ignorance or partiality, have wished to attribute to him the entire invention of the idea

* Memoires de Sully, Perefixe, &c.

† Portal Histoire d'Anatomie et Chirurgie, tom. 1.

idea of the ligature, is certainly true; but others*, more enlightened, have not attempted to support a claim which Parè himself never insisted on. He modestly avows his having met with a passage in Galen, in which it is advised to tie up the vessels from which the blood is poured. After mentioning the cruelty and disadvantages attending the use of the cautery, he proceeds, "Itaque chirurgos omnes obnixè rogatos velim, ut vetere illâ, et crudelitatis crimine obsitâ medendi viâ relictâ, hanc novam amplectantur, quam me edoctum Dei numine arbitror; non enim illam a magistris, non a quoquam alio didici, aut usurpatam unquam animadverti. Tantum apud Galenum legi, ad sanguinis fluxum sistendum, nihil esse presentius, quam vasa ipsa, quorum ductibus fit fluxio, versus radices suas, hepar nempe, et cor religare. Hoc Galeni præceptum de vinciendis, et suendis venis, et arteriis in vulneribus recentibus, ad ea quæ membrorum amputatione fiunt cum traduci posse putarem, tentavi in plerisque.*" He proceeded, at first, with caution, keeping heated irons in readiness by his side; but finding his method completely succeed, and no bleeding ensue, he bade them an eternal farewell. Moreover, in his dispute, afterwards, with Gourmelen, he examined all that had been written on this subject, and was particularly anxious to produce authorities*, to show that the use of the ligature, in hæmorrhage, was sanctioned by the ancients, and was, by no means, a cruel or a novel practice, as his countryman attempted to prove.

But to the principal point, Parè asserts that no one had ever made use of this idea, or reduced it into practice in amputations; nor has that assertion ever been proved to be false. The justly-celebrated Van Swieten*, whom no one ever accused of partiality, gives the whole honour to Parè, and mentions the great Vesalius, who flourished at the time Parè was in Italy, as still practising the ancient method*. In examining the extent of the knowledge possessed by the ancients, on this subject, he quotes the very passage produced by Chirurgicus, from Celsus; so that, without recurring

---

* Louis et Morand Memoires de l'Acad. de Chirurgie, 4to. tom. 2.

* Parai Opera Fol. Francofurti, 1594, page 373.

* He quotes Hippocrates, Celsus, Galen, Avicenna, Vigo, Guy de Chauliac, Vesalius, and many others.

* Van Swieten Comment. in Boerhaave Aphor. 4to. Lugd. Batav. tom. 1. page 348 et 823; see also Friend's History of Physic, vol. 1.

* Vesalius died in 1564. Portal, tom. 1, page 398.

ring to the father of surgery, this passage might have been met with in a book which is almost in every one's hands. Van Swieten proceeds to mention a supposed improvement on the method of Parè, introduced by Dionis; but the original practice was at last universally allowed to be the best, and has continued to be so, even to the present day; but so strong are established rules, that, many years after the death of Parè, the cautery was still employed by surgeons of the first eminence*.

By this sketch, I think I have shown that Chirurgicus was, by no means, master of the subject on which he professes to determine; and I will not tire you by producing any further evidence, to corroborate what is already so firmly established. It might perhaps be a useful employment, to examine more particularly into what has been written on this subject; and I am fully confident that, after the closest inquiry, when it is considered also that the circulation of the blood was at that time unknown, and that for a cruel operation, he substituted a safe and infinitely easier one, it will be freely allowed, that the honour given to Parè was not unjustly bestowed.

TIRO.

